# Negative Beliefs About Working with Health Problems and Support at Work as Predictors for Return to Work for People Struggling with Common Mental Disorders

**DOI:** 10.1007/s10926-024-10243-6

**Published:** 2024-10-25

**Authors:** Marianne Tranberg Bjørndal, Kristian Pihl Frederiksen, Ragne Gunnarsdatter Hole Gjengedal, Bente Bull-Hansen, Kåre Osnes, Marit Hannisdal, Odin Hjemdal

**Affiliations:** 1 Division of Mental Health and Substance Abuse, Diakonhjemmet Hospital, Postboks 23 Vinderen, 0319 Oslo, Norway; 2 Department of Psychology, Norwegian University of Science and Technology, NO-7491 Trondheim, Norway

**Keywords:** Predictors for return to work, Negative beliefs about work and health, Support at work, Sick leave, Common mental disorders

## Abstract

**Purpose:**

The purpose of this study was to investigate predictors for return to work for people struggling with common mental disorders on sick leave or at risk of sick leave. The first aim of this study was to evaluate the psychometric properties of a set of statements exploring different conditions at the workplace and assumptions about working with health problems, by investigating the factor structure, reliability and construct validity of these statements. The second aim of this study was to investigate the predictive value of the identified factors.

**Methods:**

A total of 797 patients from an outpatient mental health clinic were included in a naturalistic observational study. The study design was longitudinal. The participants filled out self-report questionnaires pre- and post-treatment.

**Results:**

A principal component factor analysis with a varimax rotation identified two factors, *Negative beliefs about working with health problems* and *Support at work*, displaying high internal consistency, 0.83 and 0.84, respectively. Separately, both factors were significant predictors of full return to work after treatment. The final multivariable analysis including both factors left *Negative beliefs about working with health problems* as a significant predictor explaining unique variance.

**Conclusions:**

*Negative beliefs about working with health problems* and *Support at work* are important predictors for work status after treatment and should therefore be addressed during treatment for common mental disorders to assist people return to work.

## Introduction

Mental health problems represent a great challenge for society. In addition to individual suffering and burden, mental health problems lead to very large costs for social and health care systems. Across the EU countries, costs related to mental ill-health are estimated to be EUR 600 billion per year [1]. Sick leave due to common mental disorders (CMD) such as depression and anxiety is increasing, and CMD is affecting one in five workers at any given time [2]. Understanding what contributes to and possibly predicting return to work (RTW) for those with CMD may contribute to reducing this societal challenge [3–6]. It is therefore important to investigate factors related to the individuals’ workplace and their own beliefs about work and health which could be potential predictors for RTW.

Work is important for most people struggling with depression and anxiety as it can contribute by giving meaning, a safer economy and a place of belonging [7]. CMD is associated with reduced work productivity [8], reduced work functioning [9] and increased risk of sick leave even after symptom remission [10, 11]. In addition, individuals with CMD typically find it more difficult to take part in social settings, and more frequently tend to avoid tasks that may increase symptoms. This may also include avoiding work settings. For people with CMD, returning to work is a complex matter in which factors related to both the individuals’ coping with external factors concerning the workplace and their mental health symptoms, play a role.

Identifying predictors for RTW for CMD are important in order to increase treatment effect and specificity regarding RTW. The low number of studies with comparable potential predictors makes it difficult to find robust predictors across studies [12–14]. Previous studies of predictors for RTW have shown somewhat scattered and conflicting results for individuals with CMD. An overview by de Vries and colleagues [13] investigating prognostic factors of sickness absence indicated that previous episodes of CMD, higher symptom severity, previous absenteeism, co-morbidity, female gender, lower educational level, smoking behaviour and low perceived general health are predictors of sickness absence [13]. De Vries et al. also found that lower symptom severity, no previous absenteeism, younger age and positive expectations concerning RTW predicted earlier RTW. Also, relevant determinants for sickness absence were job demands and control, job strain, and organisational (in)justice [13]. Another recent review found that older age, being male and higher symptom scores were predictors decreasing the probability of RTW [14]. Importantly, there seems to be a gap in the literature with regards to the role of workplace factors such as social support and autonomy. Although several qualitative studies have highlighted the importance of social support [15, 16], workload [17–19] and autonomy [16, 20] in relation to RTW for CMD samples, the quantitative data for these variables have been sparse [13, 14]. Investigation of workplace factors such as social support, work load and autonomy as predictors of RTW is therefore warranted.

There is substantial comorbidity between CMD, chronic pain and fatigue [21–23], which has led some researchers to investigate RTW-interventions for people on sick leave independent of diagnosis [24], as well as taking inspiration from the research field of RTW for pain when designing RTW-interventions for CMD (e.g. [25]). Self-efficacy and fear-avoidance of pain have been described as the most important beliefs related to lower back pain, where a high level of disability is associated with low self-efficacy and high fear-avoidance behavior [26]. Fear-avoidance theory proposes that patients alter movement and activity levels due to fear of potential pain or fear that the movement will make their condition worse. This is based on their previous experience of how physical activity increases sensation of pain, which in turn can lead to fear-avoidance beliefs. This belief can also lead to different coping strategies such as avoidance of activities. Such avoidance behaviour may maintain pain-related fear due to lack of corrective experiences, which subsequently often leads to restricting or avoidance of activities and interference with valued life activities*.* Fear-avoidance beliefs have been researched extensively and found to be predictive of RTW for a broader population of health problems [27]. The fear-avoidance beliefs questionnaire (FABQ) [28] is widely used for measuring fear-avoidance behaviour, although it has been criticised for measuring expectations regarding RTW rather than fear [29]. Beliefs about avoidance may also be relevant for patients with CMD in relation to work, e.g. they may have beliefs about work potentially making them worse. However, there are few studies looking specifically at beliefs about work and fear-avoidance in relation to RTW for CMD. We are therefore interested in whether negative assumptions about working with general health problems could be predictive of RTW for CMD populations, as avoidance is a common strategy for people suffering from CMD. Examples of avoidance in a work setting may be avoiding work tasks, avoiding people or work all together.

The RTW-process is often complex and consists of an interplay between psychological factors and external, work-related factors. To fill a gap in the knowledge, the current study investigated this using newly developed or adapted statements exploring different conditions at the workplace and assumptions about working with health problems. These were expected to be of importance for individuals struggling with CMD and on sick leave or at risk of being sick-listed. The first aim of this study was to evaluate the psychometric properties of this questionnaire by investigating the factor structure, reliability and construct validity. The second aim of this study was to investigate the predictive value when it comes to RTW for the identified factors.

## Methods

### Study Context

Data were collected as part of a naturalistic observational study in the project “The Norwegian studies of psychological treatments and work (NOR-WORK)” at an outpatient clinic at Diakonhjemmet Hospital in Oslo, Norway. The clinic is part of the specialised health care system in Norway and offers cognitive or metacognitive treatment with a work-focus for patients with CMD on sick leave or at risk of becoming sick-listed. Patients were referred by their general practitioners and were between the age of 18 and 65. After referral the patients underwent a screening session, where they were assessed for eligibility for treatment. For the diagnostic evaluation the Mini International Neuropsychiatric Interview version 6.0.0 [30] was used, and diagnoses were given in accordance with the International Classification of Diseases-10. Exclusion criteria for being treated at the clinic were severe mental disorders such as bipolar disorder, cluster A or B personality disorder or psychosis, high risk of suicide or substance abuse. These patients were referred for treatment elsewhere. All patients who were offered treatment were given a new session within eight to 12 weeks, depending on the waiting time at the clinic. This is in line with national guidelines for maximum waiting times for patients. As part of the treatment all patients filled out questionnaires. The data for those giving consent were included in the ongoing study. No changes were made in the treatment plan or process for the included patients.

### Participants

A total of 889 participants consented to be included in the study from the 15th of May 2017 till the end of December 2019. The study sample, for the factor analysis, consisted of participants that had complete data for the relevant questionnaire at the screening session (*n* = 797). For the logistical regression analyses, only participants that had filled out the questionnaire at the end of the treatment (*n* = 387), as well as at the screening, were included.

### The Treatment

The treatment was individualised and consisted of protocol-based cognitive behavioural therapy [31] or metacognitive therapy [32] and work interventions. The treatment was short-term and the average number of sessions were 10.2 (SD = 4), each session lasting 45 min. The work interventions were developed at the clinic and based on Lagerveld et al. [12]. These interventions included psychoeducation about mental health and work, analysis of the patients’ work situation and need for adjustments, making a RTW plan, discussing barriers for RTW, developing an information strategy towards the workplace and cooperation with the patients’ general practitioner [33].

### Measures/Instruments

All data were collected through self-report questionnaires with the same questionnaire filled out pre- and post-treatment. The background variables included in this study were current work status, sex, age and education.

The variable for education was made into a dichotomous variable. The variable was divided into higher education defined as more than four years at University/college and lower education including primary years, high school, and University/college less than four years. Work status was measured by asking about current income situations at pre- and post-treatment. The variable was coded into a dichotomous variable; working or on full or partial sick leave.

#### Predictors of Return to Work

To investigate if different work-related aspects and negative beliefs were predictors for RTW, 14 statements were used (see Table 3 for an overview of the statements). Statements one to nine are related to aspects at work, and statements 10 to 14 covers negative beliefs about work and health. The statements were scored from “totally disagree = 0” to “totally agree = 5” on a 6-point Likert scale. The first five statements assessed aspects of the work situation such as time pressure, engagement in repetitive tasks, adequate training, freedom to decide how to perform tasks and the ability to influence one’s own work. A high score on time pressure would indicate having time pressure at work. Further, statements seven to nine evaluated how the supervisor treats you, supports you in difficult situations and how supervisor and colleagues support you if you are feeling poorly. A high score on these statements indicated good social support. These nine statements were developed at the clinic based on the job-demand-control-support model by Karasek and Theorell [34].

To measure negative beliefs about work and health, five questions from the Fear-avoidance belief questionnaire (FABQ) [28] estimated to be of relevance for CMD were chosen. These questions were selected by psychologists at the clinic with extensive experience. The FABQ was developed by Waddel et al. aiming at measuring patients lower back pain, and their beliefs about how physical activity and work affected their pain [28]. The questions from the FABQ were adapted to CMD and expanded to include health-related issues rather than pain specifically. The statements were selected based on relevance for the current sample and included the following; if the current health problems are work related, if work aggravates the health problems, if one should not or cannot perform ordinary work tasks with the current health problems, and if one will ever return to previous level of functioning. Here a higher score indicated more negative beliefs about working with health problems.

#### Symptoms of Depression and Anxiety

Beck Depression Inventory—II (BDI-II) [35] was used to measure symptoms of depression. It consists of 21 items, scored from 0 to 3. Each item is a list of four statements arranged in increasing severity about a particular symptom of depression. The scores are summed up and higher scores indicate higher levels of symptoms. The Cronbach’s alpha for BDI-II of this study was 0.86.

Beck Anxiety Inventory (BAI) [36] was used to measure symptoms of anxiety. It consists of 21 items rated on a scale from 0 to 3. Each item is descriptive of subjective, somatic or panic-related symptoms of anxiety. As with BDI-II, higher scores indicate higher levels of symptoms. The Cronbach’s alpha for BAI of this study was 0.89.

### Statistical Analyses

Data were analysed using StataIC 18.0. The suitability of performing a principal component factor analysis was investigated by performing a Kaiser-Meyer-Oklin test. The 14 statements believed to be predictors of RTW (Table 3) were subjected to a principal component factor analysis with a varimax rotation. This was done in order to possibly reduce the number of items and to investigate the underlying factor structure by identifying latent constructs and central items. Only participants with complete data for the relevant questionnaire at the screening session (*n* = 797) were included. This was to maximise the statistical foundation of the study. Criteria for identifying factors were that each of them should contain no less than three items, the items should have a clear main loading on one factor, with no factor side loadings > 0.3, and factors should have a Cronbach’s alpha between 0.7 and 0.9.

For the exploration of predictive value of the factors on work status post-treatment, multivariate logistical regression analyses were undertaken with participants that had filled out the questionnaire at the screening session and at the end of the treatment (*n* = 387). The identified factors were used as independent variables, with sex, age, education, work status, BDI-II and BAI as control variables. Missing data for individual items on BDI-II and BAI were replaced by weighted means [37]. Pairwise correlation analyses (Pearson) were conducted to investigate correlations between the identified factors and BDI-II and BAI.

To investigate if there were significant differences between those who had filled out the questionnaires at pre-, and post-treatment with those who had only filled out pre-treatment, we used an independent t-test to check for differences in age and symptoms of anxiety and depression in the two groups. For sex and education, we conducted chi-square tests.

## Results

### Descriptive Statistics

Table 1 displays an overview of the participants at baseline included in the study. There was a larger proportion of women (69.6%) than men (30.4%). The mean age for the participants was 36.9 years (SD = 10.4). The BDI-II and BAI scores of the participants indicate moderate symptoms of depression and anxiety at baseline. Table 2 displays pre- and post-information about the participants included in the logistical regression analysis.

**Table 1:** Participants characteristics at baseline (*n* = 797)

	*n*	%	Mean	SD
Sex				
Female	555	69.6		
Male	242	30.4		
Age, years			36.9	10.4
Education				
Lower education	453	56.9		
Higher education	342	43.0		
Work status				
Full or partial sick leave	365	45.8		
Working	432	54.2		
BDI-II^a^	793		26	8.9
BDI-II^a^ ≥ 14	687	86.6		
BAI^b^	796		18.8	10.0
BAI^b^ ≥ 15	486	61		

^a^BDI−II Beck Depression Inventory−second edition. A score of ≥14 indicate clinically significant level of depression

^b^BAI Beck Anxiety Inventory. A score of ≥ 15 indicate clinically significant level of anxiety

**Table 2:** Work status, BDI-II^a^ and BAI^b^ pre- and post-treatment (*n* = 378)

	Pre-treatment				Post-treatment			
	*n*	%	Mean	SD	*n*	%	Mean	SD
Work status								
Full or partial sick leave	168	49			104	30.4		
Working	175	51			238	69.6		
BDI-II^a^	342		26.6	9	339		10.4	9.2
BAI^b^	343		18.6	10.3	339		6.6	6.6

^a^BDI−II Beck Depression Inventory−second edition

^b^BAI Beck Anxiety Inventory

### Factor Structure

The Kaiser-Meyer-Oklin test showed a value of 0.81, which exceeded the recommended minimum value of 0.6 and thereby supported conducting a principal component factor analysis. This analysis revealed that there were four factors with eigenvalues exceeding 1, explaining 0.62 of the total variances. Inspection of the scree plot showed a sharp decrease after the first factor and indicated a slightly sharper brake after the third factor. The rotated factor loadings revealed that there were many items with loadings of 0.3 and above, see Table 3. To optimise construct clarity the items with loadings above 0.3 on several factors were excluded. This resulted in factor one including four items, factor two three items, and factor three and four with only two items. As the pre-set criterion was to include a minimum of three items, two factors remained, explaining 0.43 of the total variances. This excluded themes related to the workplace such as time pressure and autonomy. The identified factors were labelled; *“Support at work”*, and; *“Negative beliefs about working with health problems”*. The factor loadings on these items were high, ranging from 0.71 to 0.87 (Table 3). The Cronbach’s alpha indicated high internal consistency for the two factors, 0.84 and 0.83, respectively. The included items for each factor can be seen in Table 3.

**Table 3:** Principal component factor analysis with rotated factor loadings^1^, eigenvalue and unique variances (*n* = 797)

Variable	Factor1^a^	Factor2^b^	Factor3	Factor4	Uniqueness
1. I am under constant time pressure in my job due to a heavy workload		0.33		0.66	0.45
2. My job involves that I have to perform many repetitive tasks			0.76		0.41
3. I have tasks that I lack adequate training to perform				0.64	0.54
4. I have little freedom to decide how to perform my job			0.69		0.38
5. I have a good opportunity to influence decisions that affect my work situation	0.49		0.49		0.50
6. My supervisor treats me fairly	**0.77**^ϯ^				0.34
7. I get good support in difficult situations at work	**0.85**^ϯ^				0.26
8. If I were to feel poorly, I think my supervisor would be supportive	**0.87**^ϯ^				0.22
9. If I were to feel poorly, I think my colleagues would be supportive	**0.71**^ϯ^				0.49
10. My health problems are work related	0.31	0.63			0.41
11. My work aggravates my health problems		**0.75**^ϯ^			0.31
12. I should not perform my ordinary work tasks with my current health problems		**0.86**^ϯ^			0.24
13. I cannot perform my ordinary work until my health problems are dealt with		**0.85**^ϯ^			0.27
14. I do not think I will ever be able to return to my previous level of functioning		0.47		−0.46	0.55
Eigenvalue	**4.39**	**1.94**	**1.21**	**1.12**	
Cronbach alfa	**0.84**	**0.83**			

^1^All factor loadings < 0.3 were omitted from the table

^ϯ^Items included in factors

^a^Factor 1: *Support at work*

^b^Factor 2: *Negative beliefs about working with health problems*

### Correlation and Between Group Differences

Correlations between the two factors, BDI-II, and BAI were small and significant except between BAI and *Support at work* which was not significant. The direction was as expected and can be seen in Table 4. The chi-square test for independence indicated no significant differences in sex but indicated significantly higher education level in the group with pre- and post-treatment data than the group with only pre-treatment data, *χ*^*2*^ (1, 798) = 4.8, *p* = 0.03^*^, phi = 0.08. The independent sample t-test found no significant differences in age or in symptoms of anxiety and depression.

**Table 4:** Correlation between Support at work, Negative beliefs about working with health problems, BDI-II and BAI (*n* = 793)

	Negative beliefs about working with health problems	Support at work	BDI-II
Support at work	−0.31^***^		
BDI-II^a^	0.31^***^	−0.22^***^	
BAI^b^	0.14^***^	−0.07	0.38^***^

^a^BDI−II Beck Depression Inventory−second edition

^b^BAI Beck Anxiety Inventory

^***^p<0.001

### Predictors of RTW

We investigated if the factor scores of *Support at work* and *Negative beliefs about working with health problems* at pre-treatment predicted work status at post-treatment using two separate multivariate logistical regression analyses, see Table 5. We controlled for sex, age, education, work status and symptoms of anxiety and depression. The results indicated that sex, age, education and symptom scores at pre-treatment did not significantly predict work status at the end of the treatment. As expected, work status at pre-treatment predicted work status post-treatment. Separately, the two factors were significant predictors of full RTW at the end of the treatment. In the final multivariable analysis, we entered both factors in the same step in a model to investigate unique variance. Only the factor *Negative beliefs about working with health problems* was a significant predictor which explained unique variance.

**Table 5:** Logistical regression analysis of predictors of RTW on work status post-treatment (*n* = 387)

Working post-treatment	Odds ratio	Std.Err	Z	*p* >|z|	[95% Conf.Interval]	
Sex	0.85	0.24	−0.55	0.579	0.49	1.49
Age	1.01	0.01	0.53	0.595	0.98	1.03
Education	1.16	0.3	0.59	0.558	0.71	1.91
Work status pre-treatment	5.97	1.57	6.78	0.000	3.56	10.01
BDI-II^a^	0.98	0.02	−1.56	0.119	0.95	1.01
BAI^b^	0.99	0.01	−0.73	0.468	0.97	1.02
In separate analyses						
Negative beliefs about working with health problems	0.69	0.07	−3.73	0.000	0.56	0.84
Support at work	1.27	0.14	2.27	0.023	1.03	1.57
Factors in same analysis						
Negative beliefs about working with health problems	0.7	0.07	−3.42	0.001	0.58	0.86
Support at work	1.2	0.13	1.66	0.097	0.97	1.49

^a^BDI−II Beck Depression Inventory−second edition

^b^BAI Beck Anxiety Inventory

## Discussion

The current study set out to address a knowledge gap in relation to RTW for patients undergoing treatment for CMD focusing on measuring important workplace factors such as social support and beliefs related to health and work. A principal component factor analysis identified two psychometrically sound factors, *Support at work* and *Negative beliefs about working with health problems*, which were also significant predictors for RTW after treatment when controlling for sex, age, education and symptoms. *Negative beliefs about working with health problems* is an especially important factor explaining unique variance. These factors tap into the way patients perceive the support they receive at work and their beliefs about their symptoms in relation to their work.

The exploratory factor analysis displayed a two-factor solution, which reduced the statements from 14 to seven. The psychometric properties were acceptable and support the use of these statements as a scale. A reduction of statements is beneficial as it reduces the burden and response time and when used in a clinical setting reduces the time required by the therapist for assessment. The correlation analysis of the two factors revealed a small but significant negative correlation, indicating that the two factors might be associated but can still be considered as two distinct concepts, which can be used together or independently. The validity was further supported by a small but significant positive correlation between BDI/BAI and *Negative beliefs about working with health problems.* This was as expected as higher symptoms of CMD could be associated with more negative beliefs about working with health problems. *Support at work* had a significant negative correlation with BDI but the correlation with BAI was not significant. This may indicate that perceived lower social support at work is something patients with higher levels of depressive symptoms also experience more often than patients with higher levels of anxiety symptoms. However, this should be investigated further.

The factor, *Negative beliefs about working with health problems*, significantly predicts RTW at the end of the treatment and explains unique variance. This means that the beliefs patients have about the consequences work has on their mental health at the start of the treatment influence whether they have returned to work or not at the end of the treatment. This is in line with previous findings for patients with other health problems [27] and makes sense given the substantial co-morbidity between CMD and other somatic health problems [21–23]. This is an important finding, given the fact that the likelihood of RTW in general decreases over time [38]. It could be that patients who avoid less and are less afraid of their symptoms related to work tasks, are more inclined to engage in a graded RTW in parallel with treatment. An implication could therefore be to be wary of what patients on sick leave due to CMD believe are the consequences of working with symptoms. When needed, address how the strategy of avoidance and isolation may maintain symptoms. Many patients worry about aspects related to the work situation, e.g. if they will be able to perform their tasks, what to say to colleagues, if working with symptoms can be harmful and if symptoms will increase as a consequence of work. It is also important to address such worries and explore the potential benefits of work for mental health, which may be highlighted using advantages and disadvantages analyses of working with symptoms. Work is also an arena well fit for testing assumptions, so designing behavioural experiments related to a patient’s fears, negative beliefs and worries about working with symptoms may be a highly potent approach. Although beliefs about working with symptoms have been assumed to be important for RTW and the above mentioned interventions have been implemented in work interventions previously with good results [4, 39], our study is the first that we are aware of that specifically shows the predictive value of *Negative beliefs about working with health problems* in regards to RTW for CMD.

The factor *Support at work* consists of items including being treated fairly by the supervisor, getting good support in difficult situations at work, and believing that the supervisor and colleagues will be supportive if they are feeling poorly. This means that how you are treated at work is important for returning to work after treatment. Previous research has shown inconclusive results regarding the role of social support [13, 14]. De Vries et al. [13] concluded that social support was predictive of RTW, while in the meta-analysis by Fisker et al. [14] supervisor support was not analysed due to few studies reporting supervisor support. The results from our study indicate that social support, assessed with four items, is predictive for RTW for patients with CMD that undergo psychological treatment and work interventions. This is also in line with findings from several qualitative studies on people on sick leave due to CMD that highlight the importance of social support in relation to RTW [15–17].

As seen in qualitative studies, support from coworkers and supervisors [17] is often perceived as a precursor for autonomy, workload and freedom to make decisions. One could hypothesise that having social support connected to the workplace increases the likelihood of gaining autonomy and influencing workload during and after treatment and thus potentially act as a mediator or a moderator. The qualitative study by Bjørndal et al. [17] indicated that this was the case for some of the participants who underwent the same therapy as in the current sample. They emphasised that support from their supervisor led to temporary reductions in workload, which facilitated RTW. Furthermore, they highlighted that the feeling of being in charge of their RTW-process was important.

For some patients on sick leave, low perceived social support at the beginning of the treatment can be due to several reasons like misunderstandings, low-frequent contact with supervisor or beliefs maintained by avoidance behaviour, which hinders disconfirmation of their negative evaluations. In such cases, the therapist can aid the patient by examining both psychological aspects and concrete aspects of the job situation. One study of patients in rehabilitation before RTW found that patients with high anxiety related to work topics reported more negative workplace characteristics [40]. A qualitative study of sick-listed women due to CMD by Hedlund [18] indicates that the difference between intended support and perceived pressure can be complicated, which points to the importance of communication between the employee and the workplace. By helping the patient to reduce avoidance and get in contact with the workplace, the therapy can contribute to increase the perceived social support and thus increase the likelihood of the employer making adjustments during the RTW-process. On the other hand, low perceptions of social support can also reflect a poor work environment, lack of value and respect, and bullying. This can negatively affect and cause symptoms of CMD [41], making RTW an impossible option at the current workplace. A study investigating the treatment effects of patients with CMD exposed to workplace bullying found that those who were bullied at the workplace had a less successful RTW compared to non-bullied [42]. In such cases the goal should be to early identify the negative working conditions, in order to change the goals from returning to the previous workplace, to finding a new job. The findings in our study add weight to the importance of recognising the patients’ social support early. This in order either to help the patients modify their beliefs and thereby facilitating dialogue with the workplace and in turn facilitate adjustments, or advice for changing work in cases where bullying make return to the current workplace inadvisable.

Themes like time pressure and autonomy did not turn out to be a factor in our analysis and therefore their predictive values were not analysed further. This finding in the current study could be related to at least two different issues, characteristics of the sample and the phrasing of the questions. The participants in this study are highly educated, approximately 80% have some form of higher education compared to Norway’s national average of 36.9% [43]. Their level of education may indicate that the participants have jobs with high levels of autonomy and thus relatively lower time pressure. If so, we may have a ceiling effect and the variance may be limited. Future studies could therefore investigate autonomy and workload as predictors of RTW for CMD samples in more heterogeneous samples, as reviews have pointed to insufficient evidence base for these variables [13, 14].

## Limitations

There are some limitations to this study. The results of the factor analysis are associated with the sample explored and replication is clearly needed. However, this study uses a relatively large sample size, which is a strength as larger samples often identify more robust factor solutions (e.g. [44]). Further, the sample in this study is representative for the patients referred to this outpatient clinic. This is a clinic for patients with CMD at risk of sick leave and as such the heterogeneity in e.g. referred diagnoses in other outpatient clinics in Norway is higher. A large study conducted elsewhere in Norway investigating patients treated in the specialised mental health care displayed similar clinical characteristics in relation to the severity of anxiety and depressive symptoms. The patients there had moderate levels of anxiety measured by Generalised anxiety disorder questionnaire (GAD-7) and moderate to severe levels of depression measured by Patient health questionnaire (PHQ-9) [45]. Other limitations in relation to the sample were that the participants were highly educated and there were more women than men. Therefore, future studies with more heterogeneous samples are needed.

This was a naturalistic observational study conducted at an outpatient mental health clinic which is often considered an advantage in relation to ecological validity of a study. Another strength is the longitudinal design with prediction over time as this points to predictors that may be of importance for therapy outcomes. For the logistical regression analysis, we only included participants with data at pre- and post-treatment. Therefore, we investigated if there were differences between those who had data only at pre-treatment and those who had at both time points. There were no significant differences in sex, age and symptoms of anxiety and depression, but there was a significant difference in education. Those who had filled out data at both time points had significantly higher education compared to the other group.

## Implications and Conclusions

Sick leave among patients with CMD is elevated with high individual and societal costs. Identifying predictors for RTW is thus essential. The current study found that *Support at work* is a predictor for RTW, which highlights the importance of awareness and potentially addressing social support at work for clinicians. In cases of avoidance of dialogue with the workplace and overly negative perceptions of social support, the clinician can enable communication with the workplace and promote a dialogue related to making adjustments in the work situation.

The findings in this study show that *Negative beliefs about working with health problems* could be an especially important predictor for RTW for patients with CMD. These negative beliefs can act as barriers, affecting their motivation and engagement in their RTW process. This result suggests that this is something that the clinician particularly should explore during initial assessment. By identifying and addressing these beliefs in therapy, clinicians can better support patients in their RTW process.

*Support at work* and *Negative beliefs about working with health problems* were assessed only using seven items, which highlights that these predictors can be assessed effectively with limited strain on the patients. Further studies are clearly needed to explore the replicability of the findings using other outcome measures.

## Data Availability

The participants in this study have not consented to distribution of the data outside the study conducted at the clinic and the conditions regarding confidentiality, privacy protection and data handling approved by the Data Protection Office at Diakonhjemmet Hospital. Requests can be directed to MTB at Marianne.bjorndal@diakonsyk.no.
